# A randomized open-label trial to evaluate the efficacy and safety of triple therapy with aspirin, atorvastatin, and nicorandil in hospitalised patients with SARS Cov-2 infection: A structured summary of a study protocol for a randomized controlled trial

**DOI:** 10.1186/s13063-021-05361-y

**Published:** 2021-07-15

**Authors:** Ambudhar Sharma, Charu Sharma, Sujeet Raina, Balraj Singh, Devendra Singh Dadhwal, Vinay Dogra, Swatantra Gupta, Shyam Bhandari, Vivek Sood

**Affiliations:** 1 Department of Cardiology, Dr. Rajendra Prasad Government Medical College, Tanda, Kangra, Himachal Pradesh 176001 India; 2 Department of Anaesthesia, Dr. Rajendra Prasad Government Medical College, Tanda, Kangra, Himachal Pradesh 176001 India; 3 Department of Internal Medicine, Dr. Rajendra Prasad Government Medical College, Tanda, Kangra, Himachal Pradesh 176001 India; 4 Department of Preventive and Social Medicine, Indira Gandhi Medical College, Shimla, 171001 India; 5 Department of Pulmonary Medicine, Dr. Rajendra Prasad Government Medical College, Tanda, Kangra, Himachal Pradesh 176001 India; 6 Department of Endocrinology, Dr. Rajendra Prasad Government Medical College, Tanda, Kangra, Himachal Pradesh 176001 India; 7 Department of Gastroenterology, Dr. Rajendra Prasad Government Medical College, Tanda, Kangra, Himachal Pradesh 176001 India

**Keywords:** COVID-19, Aspirin, Atorvastatin, Nicorandil, Randomised control trial, Protocol, Hospitalised patients

## Abstract

**Objectives:**

The pathophysiology of SARS-Cov-2 is characterized by inflammation, immune dysregulation, coagulopathy, and endothelial dysfunction. No single therapeutic agent can target all these pathophysiologic substrates. Moreover, the current therapies are not fully effective in reducing mortality in moderate and severe disease. Hence, we aim to evaluate the combination of drugs (aspirin, atorvastatin, and nicorandil) with anti-inflammatory, antithrombotic, immunomodulatory, and vasodilator properties as adjuvant therapy in covid- 19.

**Trial design:**

Single-centre, prospective, two-arm parallel design, open-label randomized control superiority trial.

**Participants:**

The study will be conducted at the covid centre of Dr. Rajendra Prasad Government Medical College Tanda Kangra, Himachal Pradesh, India.

All SARS-CoV-2 infected patients requiring admission to the study centre will be screened for the trial. All patients >18years who are RT-PCR/RAT positive for SARS-CoV-2 infection with pneumonia but without ARDS at presentation (presence of clinical features of dyspnoea hypoxia, fever, cough, spo2 <94% on room air and respiratory rate >24/minute) requiring hospital admission and consenting to participate in the trial will be included.

Patients with documented significant liver disease/dysfunction (AST/ALT > 240), myopathy and rhabdomyolysis (CPK > 5x normal), allergy or intolerance to statins, allergy or intolerance to aspirin, patients taking medications with significant interaction with statins, prior statin use (within 30 days), prior aspirin use (within 30 days), history of active GI bleeding in past three months, coagulopathy, thrombocytopenia (platelet count < 100000/ dl), pregnancy, active breastfeeding, patient unable to take oral or nasogastric medications, patients in altered mental status, shock, acute renal failure, acute coronary syndrome, sepsis and ARDS at presentation will be excluded.

**Intervention and comparator:**

After randomization, participants in the intervention group will receive aspirin, atorvastatin, and nicorandil (Fig. 1). Atorvastatin will be prescribed as 40 mg starting dose followed by 40 mg oral tablets once daily for ten days or till hospital discharge whichever is later. Aspirin dose will be 325 starting dose followed by 75 mg once daily for ten days or till hospital discharge whichever is later. Nicorandil will be given as 10 mg starting dose followed by 5mg twice daily ten days or till hospital discharge whichever is later. All patients in the intervention and control group will receive a standard of care for covid management as per national guidelines. All patients will receive symptomatic treatment with antipyretics, adequate hydration, anticoagulation with low molecular weight heparin, intravenous remdesivir, corticosteroids (intravenous dexamethasone for 5 days or more duration if oxygen requirement increasing or inflammatory markers are raised), and oxygen support. Patients will receive treatment for comorbid conditions as per guidelines.

**Fig. 1:**
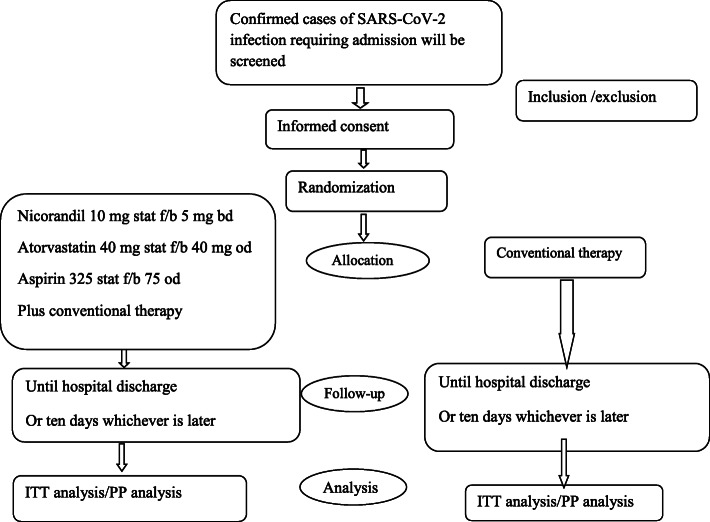
Schematic study design

**Main outcomes:**

The patients will be followed up for outcomes during the hospital stay or for ten days whichever is longer. The primary outcome will be in-hospital mortality. Any progression to ARDS, shock, acute kidney injury, impaired consciousness, length of hospital stay, length of mechanical ventilation (invasive plus non-invasive) will be secondary outcomes. Changes in serum markers (CRP, D –dimer, S ferritin) will be other secondary outcomes. The safety endpoints will be hepatotoxicity (ALT/AST > 3x ULN; hyperbilirubinemia), myalgia—muscle ache, or weakness without creatine kinase (CK) elevation, myositis—muscle symptoms with increased CK levels (3-10) ULN, rhabdomyolysis—muscle symptoms with marked CK elevation (typically substantially greater than 10 times the upper limit of normal [ULN]) and with creatinine elevation (usually with brown urine and urinary myoglobin) observed during the hospital stay.

**Randomization:**

Computer-generated block randomization will be used to randomize the participants in a 1:1 ratio to the active intervention group A (Aspirin, Atorvastatin, Nicorandil) plus conventional therapy and control group B conventional therapy only.

**Blinding (masking):**

The study will be an open-label trial.

**Numbers to be randomized (sample size):**

A total of 396 patients will participate in this study, which is randomly divided with 198 participants in each group.

**Trial status:**

The first version of the protocol was approved by the institutional ethical committee on 1^st^ February 2021, IEC /006/2021. The recruitment started on 8/4/2021 and will continue until 08/07/2021. A total of 281 patients have been enrolled till 21/5/2021.

**Trial registration:**

The trial has been prospectively registered in *Clinical Trial Registry – India* (ICMR- NIMS): CTRI/2021/04/032648 [Registered on: 8 April 2021].

**Full protocol:**

The full protocol is attached as an additional file, accessible from the Trials website (Additional file 1). In the interest in expediting dissemination of this material, the familiar formatting has been eliminated; this letter serves as a summary of the key elements of the full protocol. The study protocol has been reported under the Standard Protocol Items: Recommendations for Clinical Interventional Trials (SPIRIT) guidelines.

**Supplementary Information:**

The online version contains supplementary material available at 10.1186/s13063-021-05361-y.

## Supplementary Information

**Additional file 1.** Full Study Protocol.

## Data Availability

The datasets analysed during the current study are available from the corresponding author on reasonable request.

