# Supplementary material for: A randomized open-label trial to evaluate the efficacy and safety of triple therapy with aspirin, atorvastatin, and nicorandil in hospitalised patients with SARS Cov-2 infection: A structured summary of a study protocol for a randomized controlled trial
Source: Trials. 2021 Jul 15;22:451. doi: 10.1186/s13063-021-05361-y (PMC8280682; doi:10.1186/s13063-021-05361-y)
Supplement: Supplementary file 1 — Additional file 1. Full Study Protocol. [file 13063_2021_5361_MOESM1_ESM.docx]

**A randomized open-label trial to evaluate the efficacy and safety of triple therapy with Aspirin Atorvastatin and Nicorandil in hospitalised patients with SARS- Cov -2 infection (NAAC Trial)**

**Research Protocol (COVID-19)**

**MARCH 2021**

**DR. RPGMC TANDA DR AMBUDHAR SHARMA**

**Table of contents**

Study title ……………………………………………………………………………………...1

Principal investigator…………………………………………………………………………..3

List of abbreviations………………………………………………………………………....4-5

Introduction………………………………………………………………………………….6-7

Review of literature………………………………………………………………………...8-13

Objectives…………………………………………………………………………………….13

Trial design…………………………………………………………………………………...13

Site of study…………………………………………………………………………………..14

Eligibility criteria……………………………………………………………………………..14

Inclusion criteria……………………………………………………………………………...14

Exclusion criteria……………………………………………………………………………..14

Investigation……………………………………………………………………………....14-15

Randomization……………………………………………………………………………….15

Blinding………………………………………………………………………………………15

Sample size…………………………………………………………………………………...15

Outcome and measurement…………………………………………………………………..16

Primary outcome……………………………………………………………………………..16

Secondary outcome…………………………………………………………………………..16

Safety endpoints……………………………………………………………………………...16

Intervention groups…………………………………………………………………………...17

Intervention group……………………………………………………………………………17

Control group…………………………………………………………………………………17

Follow up……………………………………………………………………………………..17

Statistical analysis…………………………………………………………………………….18

References………………………………………………………………………………...19-22

Appendix I………………………………………………………………………………...23-26

Appendix II……………………………………………………………………………….27-33

**Principal investigator**

Dr. Ambudhar Sharma, Assistant Professor Cardiology, Room no 107, 1st floor super specialty block, Dr. Rajendra Prasad Government Medical College, Tanda Kangra, India 176001.

Phone no: 9418048268

Email ID: ambudhar414@gmail.com

**List of abbreviations**

ACE2 Angiotensin converting enzyme2

ALT Alanine transaminase

ARDS Acute respiratory distress syndrome

ASA Acetyl salicylic acid

AST Aspartate transaminase

BARC British academic research consortium

BNP Brain natriuretic peptide

CAD Coronary artery disease

CKD Chronic kidney disease

COPD Chronic obstructive airway disease

COVID -19 Coronavirus disease of 2019

CPAP Continuous positive airway pressure

CPK Creatin phosphokinase

CRP Creactive protein

CVA Cerebrovascular accident

DBP Diastolic blood pressure

DM Diabetes mellitus

HFNC High flow nasal cannula

HIV Human immunodeficiency virus

HPAECs Human pulmonary artery endothelial cells

HTN Hypertension

iNOS Inducible nitric oxide synthase

ITT Intention to treat

LDH Lactate dehydrogenase

LFT Liver function test

NF-Κb Nuclear factor -kb

NO Nitric oxide

NRM Non rebreathing mask

OR Odds ratio

PAD Peripheral arterial disease

PGE2 Prostaglandin E2

PGI2 Prostaglandin I2

PP Per protocol

RAT Rapid antigen test

RFT Renal function test

RNA Ribonucleic acid

RT-PCR Reverse Transcription Polymerase Chain Reaction

SARS-CoV-2 Severe acute respiratory syndrome coronavirus 2

SBP Systolic blood pressure

TGF-β Transforming growth factor -beta

TXA2 Thromboxane A2

ULN Upper limit of normal

VTE Venous thromboembolism

WHO World health organization

**Introduction**

COVID-19 has presented a major threat to public health worldwide. The case fatality rate of COVID-19 is 2%–3%, but the pandemic associated with COVID-19 has been far more severe. SARS-CoV-2 is highly contagious and most individuals within the population are susceptible to infection(1).

SARS-CoV-2 is a single-stranded RNA virus (2). The viral infection is cytopathic to human cells, by binding to membrane-bound angiotensin-converting enzyme 2 (ACE2). ACE2 is expressed abundantly on vascular endothelial cells. The endothelial dysfunction is associated with vasoconstriction, inflammation, permeability, and coagulation(3). The immune-mediated injury may play a critical role in the pathogenesis of COVID-19. In severe forms of COVID-19, the inflammatory cascade may lead to a cytokine storm. The cytokine storm is believed to be a key factor driving both ARDS and extra-pulmonary organ failure (1)

COVID-19 represents a spectrum of clinical manifestations that typically include fever, dry cough, myalgia, weakness, and fatigue, often with pulmonary involvement (1). The majority (81%) of patients had mild manifestations, 14% had severe manifestations, and 5% had critical manifestations. Older adults and those with comorbidities are believed to be at an elevated risk of complications (2). The complications of COVID-19 include pneumonia (75%), acute respiratory distress syndrome (15%), acute liver injury, acute cardiac injury, prothrombotic coagulopathy, acute kidney injury, neurologic manifestations, including impaired consciousness and acute cerebrovascular disease, and shock (4). The disease is associated with lymphopenia, elevated inflammatory markers, and abnormal coagulation parameters(3).

Different treatment modalities might likely have different efficacies at different stages of illness and in different manifestations of the disease. Viral inhibition would be expected to be most effective early in infection, while, in hospitalized patients, immunomodulatory agents may be useful to prevent disease progression and anticoagulants may be useful to prevent thromboembolic complications. Various drugs like antivirals, antibodies, anti-inflammatory agents, targeted immunomodulatory therapies, anticoagulants, and antifibrotics are being evaluated for the management of COVID-19 with limited success so far(4).

The current therapy for COVID-19 involves oxygen support, steroids like dexamethasone, anticoagulation with low molecular weight heparin, and antivirals like remdesivir. However, even with these therapeutic agents mortality is still higher in moderate and severe disease. In patients hospitalized with COVID-19, only dexamethasone has been found to reduce the mortality (5). These therapeutic agents or regimens are not either fully effective or sufficient to combat the complete pathophysiology of the disease.

There is a need for a drug or combination of drugs that can target every component of the pathophysiology of COVID-19. Such therapeutic regimens should be easily available, low cost, and with an established safety profile. There is no single agent which can target all these pathophysiologic substrates. Hence, we aim to evaluate the combination of commonly used cardiac medication (Aspirin, Atorvastatin, and Nicorandil) with anti-inflammatory, antithrombotic, immunomodulatory, and vasodilator properties as adjuvant therapy in covid- 19. Moreover, these drugs are already extensively used, well-tolerated, and having a good safety profile in the human population.

**Review of literature**

**Aspirin**

Aspirin is currently the most widely used drug worldwide since 1897. The benefit of aspirin treatment is now evident for acute coronary syndromes and secondary cardiovascular prevention(6). Aspirin is a typical non-steroidal anti-inflammatory drug with strong anti-inflammatory, anti-thrombotic, and analgesic pharmacological effects. However, prophylactic use of low-dose aspirin is currently controversial in patients with COVID-19(7).

Alveolar capillary micro thrombosis is thought to contribute to the severe lung injury and hypoxemia that occurs in COVID-19 patients. A prior study suggested that systemic anticoagulation reduces mortality in mechanically ventilated COVID-19 patients. In ARDS, aspirin has been studied with mixed results, where some studies have demonstrated benefit and others have not(8).

**Acetylsalicylic Acid as an Anti‑Inflammatory Drug**

ASA exerts its anti-inflammatory effects mostly as a non-selective inhibitor of cyclo-oxygenase (COX-1 and COX-2) enzymes, which are involved in the production of important mediators, including PGs and thromboxane A2 (TXA2). Additionally, ASA can increase acetylation of histone proteins, regulating gene expression. It inhibits virus replication by inhibiting prostaglandin E2 (PGE2) in macrophages and up-regulation of type I interferon production(9).

**Acetylsalicylic Acid as an Anti‑Thrombotic Drug**

Low doses of ASA (e.g., 75–100 mg/day) are sufficient to irreversibly acetylate Ser 530 of COX-1, thus preferentially inhibiting platelet generation of TXA2, a potent vasoconstrictor, and stimulator of platelet reactivity. Reduction of production of PGI2 at higher doses leads to reduced efficacy as antithrombotic. Significant inhibition of platelet activation and aggregation is achieved rapidly by using higher ASA doses ≥ 300 mg followed by lower doses of 75–100 mg daily (10).

Additional mechanisms of ASA-induced effects include down-regulation of inducible nitric oxide synthase (iNOS), oxidative phosphorylation uncoupling, and increased permeability in mitochondria. The pooled results of the Warfarin and Aspirin (WARFASA) and the Aspirin to Prevent Recurrent Venous Thromboembolism (ASPIRE) trials showed that in patients with a first unprovoked venous thromboembolism, ASA reduced the risk of thrombotic event recurrence by 32% (10).

**Acetylsalicylic Acid as an Antiviral Drug**

ASA has significant antiviral activity against several other RNA viruses, including influenza A H1N1 virus, human rhinoviruses, and coxsackievirus subtype A9 (10). Acetylsalicylic acid (ASA), inhibit NF-κB, leading to subsequent inhibition of viral replication (11).

COVID-19 positive Veterans Health Administration patients with active aspirin prescriptions have a significantly decreased risk of mortality as indicated by unadjusted odds ratios of 0.68 (95% CI of0.57–0.80) at 14 days, and 0.68 (95% CI of 0.59–0.77) at 30 days after diagnosis (12).

A retrospective, observational cohort study of adult patients admitted with COVID-19 to multiple hospitals in the United States between March 2020 and July 2020 showed that Aspirin use had a crude association with less mechanical ventilation (35.7% aspirin versus 48.4% non-aspirin, P = .03) and ICU admission (38.8% aspirin versus 51.0% nonaspirin, P = .04), but no crude association with in-hospital mortality (26.5% aspirin versus 23.2% nonaspirin, P = .51). Aspirin use may be associated with improved outcomes in hospitalized COVID- 19 patients(13).

**Potential Role of Acetylsalicylic Acid in COVID‑19**

Aspirin may be useful for the relief of specific symptoms of COVID-19, due to its analgesic and antipyretic effects. it may exert anti-inflammatory, antithrombotic, and antiviral effects, inhibiting the pathophysiological processes leading to the most severe clinical manifestations of COVID-19. Hence, ASA might reasonably be a therapeutic candidate to be tested in COVID-19. Timing of ASA initiation, dosing, duration of treatment, and subgroups of COVID-19 patients that can benefit most from ASA needs to be established.

**Statin**

Statins exert pleiotropic effects on inflammation and oxidative stress, contributing to their beneficial impact on cardiovascular diseases. Statins modulate the immune response at different levels, including immune cell adhesion and migration, antigen presentation, and cytokine production. Statins reduce reactive oxygen species and increasing antioxidants.

Infection with SARS-CoV results in increased induction of the myeloid differentiation primary response 88 (MyD88) gene, which activates the nuclear factor (NF)-kB pathway and induces inflammation. Statins inhibit the MyD88 pathway and tend to preserve MyD88 levels during hypoxia and under stress, which may confer a protective effect in COVID-19 patients (14).

Large observational studies have reported the effectiveness of statin treatment in reducing influenza-related hospitalizations and deaths. An association between outpatient statin use and reduction in disease severity among patients hospitalized during the 2009 H1N1 pandemic has also been demonstrated.

Statins also interfere with ACE2 signaling. After initial entry through ACE2, SARS-CoV-2 down-regulates ACE2 expression, and causing an unopposed angiotensin II accumulation, leading to organ injury. Statins are known to up-regulate ACE2. An increase in ACE2 might prove beneficial for COVID-19 patients (15). A large analysis from an epicentre of the COVID-19 pandemic demonstrated that antecedent statin use was associated with significantly lower rates of in-hospital mortality within 30 days(16).

In this Danish nationwide cohort study, recent statin exposure in patients with COVID-19 infection was not associated with an increased or decreased risk of all-cause mortality or severe infection(17). Statins might mitigate the effects of COVID-19 infection in selected patients based on its associated coagulopathy, endothelial dysfunction, and dysregulated inflammation (18).

A meta-analysis of 9 studies (case series and retrospective cohort studies), with a total of 3449 patients were included in the analysis. The meta-analysis showed that statin use did not improve severity outcome [OR 1.64 (95% CI 0.51e5.23), p ¼ 0.41, I2 ¼ 93%, random-effect modelling] nor mortality rate from COVID-19 infection [OR 0.78 (95% CI 0.50e1.21), p ¼ 0.26, I2 ¼ 0%, fixed-effect modelling]. This study has several limitations. Analysis was limited to retrospective studies only and confounding conditions like comorbidities were not considered (19). Further randomized studies are needed to establish the role of statins in patients with COVID-19.

**Nicorandil**

Nicorandil (N-[2-hydroxyethyl]-nicotinamide nitrate) is a therapeutic agent used clinically for the treatment of angina. Nicorandil acts by increasing nitric oxide (NO) availability and by opening ATP-sensitive K channels (K+ ATP). NO can attenuate the viral receptor (ACE2) interaction through inducing morphological changes in the viral spike (S) protein and may inhibit viral replication through diminishing viral RNA production (20).

**Inflammation and Oxidative stress and modulation by nicorandil**

Nicorandil aborted inflammatory cytokine formation and suppressed apoptosis. Nicorandil inhibits superoxide anion production by the activated neutrophils. The immunomodulatory effect of nicorandil and lung tissue protection against apoptosis is reflected in improved arterial oxygen saturation and oxygen partial pressure. Nicorandil causes bronchodilation and reduces vascular permeability. NO can inhibit the replication cycle of SARS-CoV(20).

Nicorandil can abort the inflammatory process by suppressing monocyte-endothelial adhesion. Nicorandil protected HPAECs from hypoxia-induced apoptosis. Nicorandil has an anti-apoptotic effect through activation of mitoKATP channels and increased eNOS expression, with subsequent inhibition of the NF-κB pathway and the mitochondrial apoptotic pathway(21).

**Anti -fibrotic potential of nicorandil**

The SARS-CoV2 infection has a high tendency for pulmonary parenchymal and interstitial fibrosis(22). The beneficial actions of nicorandil are signaled by a decrease in the profibrotic marker, transforming growth factor-β (TGF-β). Immunohistochemical examination revealed that nicorandil-treated rats exhibited significant diminutions in protein expression levels of transforming growth factor beta-1(TGF-β1) and inducible nitric oxide synthase (iNOS) and enhanced pulmonary protein expression of endothelial NOS (eNOS)(23).

**Anti-Coagulant effect of nicorandil**

NO inhibits platelet activation and limits endothelial-leukocyte adhesion. Nicorandil protected pulmonary endothelium from the thrombus formation and induction of apoptosis, accompanied by both upregulations of endothelial NOS expression and downregulation of cleaved caspase-3 expression(24). Nicorandil prevented sirolimus-induced thrombus formation, presumably due to the reduction of reactive oxygen species (ROS) and endothelial protection(25).

Based on the modulatory role of NO on the interstitial lung thrombo-inflammation, NO can be used as adjuvant therapy. The American Food and Drug Administration (FDA) has recently granted the safety of NO-releasing drugs as supportive therapy in COVID-19 treatment.

**Cardiovascular manifestation in covid-19 and nicorandil**

The involvement of myocardial injury may be linked to the cardiac ACE2 expression. The inflammatory storm caused by SARS-CoV2 infection, respiratory dysfunction, and hypoxemia may be other precipitating factors for the COVID-19 induced cardiac injury. The myocardial dysfunction was described without evidence of obstructive coronary disease. Nicorandil prevents cardiac fibroblast proliferation. The inhibitory effect might be associated with the opening KATP channels, by interfering with the generation of ROS (26). Nicorandil protected cardiac tissues by normalization of cardiac biochemical and oxidative stress parameters and amelioration of histopathological changes(27).

**Nephroprotective effect of nicorandil**

The pathologic hallmark of COVID-19 is proximal tubular injury and loss of brush border due to direct involvement. Other factors mediating acute kidney injury, include systemic hypoxia, coagulopathy, and possible drug nephrotoxicity. Nicorandil administration significantly restored mitochondrial enzymes and oxidative phosphorylation efficacy mediated through enhanced mitoKATP channel function. Based on the various possible benefits of nicorandil therapy in the COVID-19 management, Nicorandil possesses multiple potential modulatory properties on the currently known pathogenesis of the disease(28)**.**

**Method and Materials**

**Objectives**

To test whether triple therapy with aspirin, atorvastatin, and nicorandil is superior to usual care in SARS-CoV-2 infected patients in improving outcome.

To find out the safety of triple therapy in SARS-CoV-2 infected patients.

**Study Design**: Single-centre, prospective, two-arm parallel design, open-label randomized control superiority trial.

**Place of Study**: The study will be conducted at the covid centre of Dr. Rajendra Prasad Government Medical College Tanda Kangra, Himachal Pradesh India, converted into a dedicated COVID-19 management centre since August 2020.

**Eligibility criteria**

**Inclusion criteria:**

All SARS-CoV-2 infected patients requiring admission to the study centre will be screened for the trial (figure 1). All patients >18years who are RT-PCR/RAT positive for SARS-CoV-2 infection with pneumonia but without ARDS at presentation (presence of clinical features of dyspnoea hypoxia, fever, cough, spo2 <94% on room air and respiratory rate >24/minute) requiring hospital admission and consenting to participate in the trial will be included.

**Exclusion criteria:**

Patients with documented significant liver disease/dysfunction (AST/ALT > 240), myopathy and rhabdomyolysis (CPK > 5x normal), allergy or intolerance to statins, allergy or intolerance to aspirin, patients taking the following medications: cyclosporine, HIV protease inhibitors, hepatitis C protease inhibitor, telaprevir, fibric acid derivatives (gemfibrozil), niacin, azole antifungals (itraconazole, ketoconazole) clarithromycin, and colchicine, prior statin use (within 30 days), prior aspirin use (within 30 days), history of active GI bleeding in past three months, coagulopathy, thrombocytopenia (Platelet count < 100000/ dl), pregnancy, active breastfeeding, patient unable to take oral or nasogastric medications will be excluded. The patients in altered mental status, shock, acute renal failure, acute coronary syndrome, sepsis, and ARDS at presentation will be excluded.

**Investigation**

Baseline investigations like complete blood count, liver function test, renal function test, fasting blood sugar, LDH, D-dimer, CRP, serum ferritin, BNP, Trop-I, CPK, PT/INR, procalcitonin - will be done at admission. RFT, LFT, CPK, Trop-I, BNP, D-dimer, CRP, serum ferritin will be repeated on the 5^th^ day and at the time of discharge.

**Randomization**

Enrollment eligibility will be accessed by the person admitting patients. Before assigning groups to eligible individuals to participate in the study, written informed consent will be taken by the medical officer posted in the covid centre. Block randomization technique will be used to assign patients to the standard care (control) or current intervention (intervention group). We will use a block size of four study participants during the randomization process. Allocation to intervention or control group will be done by the treating team in the covid centre. Allocation concealment is done by the treatment team.

**Blinding**

This will be an open-label study. In this study, all participants are aware of participating in the study and enroll in the study after consent. The participants will be aware of the group assigned. Patients receive aspirin, atorvastatin, nicorandil in the intervention group(A) over and above the standard of care, and patients in the control group (B) will receive standard of care only. The health care personal, data collecting officials, and those who evaluate the outcome are aware of the grouping of patients.

**Sample size**

A total sample size of 396 was calculated assuming alpha error=0.05, power 80%, death in the control group of 20% (5), the death rate in the treatment group =15%, a superiority margin of 5%, and a nonresponse rate (loss to follow up) of 10% using sample size calculator <http://riskcalc.org/samplesize/>.

**Outcome and measurement**

Data collection is done by the specially trained nursing officers of the hospital. Patients’ demographic data and clinical data including age, sex, comorbidities, baseline vital information, saturation, treatment received, type of oxygen support, baseline and follow-up investigation information, outcome, and safety endpoint information will be recorded on structured proforma.

**Primary outcomes**

The primary outcome will be in-hospital mortality.

**Secondary outcomes**

The secondary outcomes will be any progression to ARDS, shock, acute kidney injury, impaired consciousness, and length of hospital stay, length of mechanical ventilation (invasive plus non-invasive). Changes in serum markers (CRP, D –dimer, S ferritin) will be other secondary outcomes.

**Safety endpoints**

Hepatotoxicity (ALT/AST > 3x ULN; hyperbilirubinemia), myalgia—muscle ache or weakness without creatine kinase (CPK) elevation, myositis—muscle symptoms with increased CPK levels (3-10)ULN, rhabdomyolysis—muscle symptoms with marked CPK elevation (typically substantially greater than 10 times the upper limit of normal [ULN]) and with creatinine elevation (usually with brown urine and urinary myoglobin), minor Bleeding (BARC bleeding type 1 and 2 or major Bleeding (BARC bleeding type 3 –5 ) will be safety endpoints observed during the hospital stay.

**Intervention groups**

**Intervention group**

Atorvastatin will be prescribed as 40mg starting dose followed by 40 mg oral tablets once daily for ten days or till hospital discharge whichever is later. Aspirin dose will be 325 starting dose followed by 75mg once daily for ten days or till hospital discharge whichever is later. Nicorandil will be given as 10 mg starting dose followed by 5mg twice daily for ten days or till hospital discharge whichever is later. In addition, all patients will receive symptomatic treatment with antipyretics, adequate hydration, anticoagulation with low molecular weight heparin, intravenous remdesivir, corticosteroids (intravenous dexamethasone for 5 days or more duration whenever indicated, if oxygen requirement increasing or inflammatory markers are raised), and oxygen support. Patients will receive treatment for comorbid conditions as per guidelines.

**Control group**

All patients in the control group will receive symptomatic treatment with antipyretics, adequate hydration, anticoagulation with low molecular weight heparin, intravenous Remdesivir, corticosteroids (intravenous dexamethasone for 5 days or more duration whenever indicated, if oxygen requirement increasing or inflammatory markers are raised), and oxygen support. Patients will receive treatment for comorbid conditions as per guidelines.

**Follow-up**

All study participants will be prospectively followed up during the hospital stay or 10 days whichever is later. Patients will be evaluated telephonically post-discharge till ten days.

**Withdrawal from the study**

Complete and accurate follow-up is extremely important for the duration of the study. The participant, however, may decline to continue protocol-related assessments at any time however every attempt will be made to continue contact by telephone. This does not constitute withdrawal from the study. The reason for withdrawal will be documented for all participants withdrawn from the study. If the vital status is known before hospital discharge, the participant will not be considered lost to follow up.

**Statistical Analysis:**

Descriptive statistics will be used to summarize variables. All primary and additional endpoints will be analysed both on intention-to –treat (ITT) basis and on a per-protocol (PP) basis for the study. The ITT population set consists of all patients who have provided informed consent and have been randomized to a treatment group. All patients will be analysed according to the assigned treatment group, regardless of the treatment received.

The PP population set will consist of all patients who have provided informed consent and have been randomized to a treatment group, and who have received only the assigned study treatment, without any major protocol deviation. Participants who do not receive a study treatment, or who receive any treatment other than the study treatment to which they were randomized, will be excluded from the PP population. All participants will be analysed according to the treatment that they received. A supportive analysis of the primary endpoint and all secondary clinical endpoints will also be conducted in the PP population. Wilcoxon rank-sum test will be used for nonnormal distribution. Time-to-event will be compared using the Kaplan-Meier curve and log-rank test. The Cox proportional hazards model will be used to calculate the hazards ratio and 95% Confidence Interval. Safety outcomes will be compared between the groups using Chi-square or Fisher’s exact test.

**Data Recording and Record-Keeping**

All trial data will be entered by trained nursing officials on the Microsoft Excel worksheet. Participants will be provided with a unique identification number. The data received by the researchers will not include any personally identifiable information. The data will be kept for 10 years.

**Bibliography**

1. Shi Y, Wang G, Cai X peng, Deng J wen, Zheng L, Zhu H hong, et al. An overview of COVID-19. Journal of Zhejiang University: Science B. 2020;21(5):343–60.

2. Qu L, Li J, Ren H. COvid-19: The epidemiology and treatment. British Journal of Hospital Medicine. 2020;81(10):1–9.

3. Haubner B, Tanner FC, Ruschitzka F, Flammer AJ, Matthias PN. Since January 2020 Elsevier has created a COVID-19 resource centre with free information in English and Mandarin on the novel coronavirus COVID- 19 . The COVID-19 resource centre is hosted on Elsevier Connect , the company ’ s public news and information . 2020;(January).

4. Wiersinga WJ, Rhodes A, Cheng AC, Peacock SJ, Prescott HC. Pathophysiology, Transmission, Diagnosis, and Treatment of Coronavirus Disease 2019 (COVID-19): A Review. JAMA - Journal of the American Medical Association. 2020;324(8):782–93.

5. Horby P, Lim WS, Emberson J, Mafham M, Bell J, Linsell L, et al. Effect of dexamethasone in hospitalized patients with COVID-19 – Preliminary report. medRxiv. medRxiv; 2020. p. 2020.06.22.20137273.

6. Montinari MR, Minelli S, De Caterina R. The first 3500 years of aspirin history from its roots – A concise summary. Vascular Pharmacology. 2019;113:1–8.

7. Yuan S, Chen P, Li H, Chen C, Wang F, Wang DW. Mortality and pre-hospitalization use of low-dose aspirin in COVID-19 patients with coronary artery disease. Journal of Cellular and Molecular Medicine. 2020;(November):1–11.

8. Chow JH, Khanna AK, Kethireddy S, Yamane D, Levine A, Jackson AM, et al. Aspirin Use is Associated with Decreased Mechanical Ventilation, ICU Admission, and In-Hospital Mortality in Hospitalized Patients with COVID-19. Vol. Publish Ah, Anesthesia & Analgesia. 2020.

9. Mohamed-Hussein AAR, Aly KME, Ibrahim MEAA. Should aspirin be used for prophylaxis of COVID-19-induced coagulopathy? Medical Hypotheses. 2020;144:109975.

10. Bianconi V, Violi F, Fallarino F, Pignatelli P, Sahebkar A, Pirro M. Is Acetylsalicylic Acid a Safe and Potentially Useful Choice for Adult Patients with COVID-19 ? Drugs. 2020 Sep 1;80(14):1383–96.

11. Mazur I, Wurzer WJ, Ehrhardt C, Pleschka S, Puthavathana P, Silberzahn T, et al. Acetylsalicylic acid (ASA) blocks influenza virus propagation via its NF-κB-inhibiting activity. Cellular Microbiology. 2007 Jul 1;9(7):1683–94.

12. Osborne TF, Veigulis ZP, Arreola DM, Mahajan SM, Roosli E, Curtin CM. Association of mortality and aspirin prescription for COVID-19 patients at the Veterans Health Administration. PLoS ONE. 2021 Feb 1;16(2 February).

13. Chow JH, Khanna AK, Kethireddy S, Yamane D, Levine A, Jackson AM, et al. Aspirin Use Is Associated with Decreased Mechanical Ventilation, Intensive Care Unit Admission, and In-Hospital Mortality in Hospitalized Patients with Coronavirus Disease 2019. Anesthesia and Analgesia. 2021;132(4):930–41.

14. Rizk JG, Kalantar-Zadeh K, Mehra MR, Lavie CJ, Rizk Y, Forthal DN. Pharmaco-Immunomodulatory Therapy in COVID-19. Drugs. 2020;80(13):1267–92.

15. Castiglione V, Chiriacò M, Emdin M, Taddei S, Vergaro G. Statin therapy in COVID-19 infection. European heart journal Cardiovascular pharmacotherapy. 2020;6(4):258–9.

16. Gupta A, Madhavan M V, Poterucha TJ, DeFilippis EM, Hennessey JA, Redfors B, et al. Association Between Antecedent Statin Use and Decreased Mortality in Hospitalized Patients with COVID-19. Research square. 2020;1–22.

17. Butt JH, Gerds TA, Schou M, Kragholm K, Phelps M, Havers-Borgersen E, et al. Association between statin use and outcomes in patients with coronavirus disease 2019 (COVID-19): a nationwide cohort study. BMJ open. 2020;10(12):e044421.

18. Cheah K, Lee H, Sewa DW, Phua GC. Since January 2020 Elsevier has created a COVID-19 resource centre with free information in English and Mandarin on the novel coronavirus COVID- 19 . The COVID-19 resource centre is hosted on Elsevier Connect , the company ’ s public news and information . 2020;(January).

19. Lee KCH, Sewa DW, Phua GC. Potential role of statins in COVID-19. Vol. 96, International Journal of Infectious Diseases. Elsevier B.V.; 2020. p. 615–7.

20. Åkerström S, Gunalan V, Keng CT, Tan YJ, Mirazimi A. Dual effect of nitric oxide on SARS-CoV replication: Viral RNA production and palmitoylation of the S protein are affected. Virology. 2009;395(1):1–9.

21. Wang H, Zuo X, Wang Q, Yu Y, Xie L, Wang H, et al. Nicorandil inhibits hypoxia-induced apoptosis in human pulmonary artery endothelial cells through activation of mitoKATP and regulation of eNOS and the NF-κB pathway. International Journal of Molecular Medicine. 2013;32(1):187–94.

22. Ye Z, Zhang Y, Wang Y, Huang Z, Song B. Chest CT manifestations of new coronavirus disease 2019 (COVID-19): a pictorial review. European Radiology. 2020;30(8):4381–9.

23. Kseibati MO, Shehatou GSG, Sharawy MH, Eladl AE, Salem HA. Nicorandil ameliorates bleomycin-induced pulmonary fibrosis in rats through modulating eNOS, iNOS, TXNIP and HIF-1α levels. Life Sciences. 2020 Apr 1;246.

24. Sahara M, Sata M, Morita T, Hirata Y, Nagai R. Nicorandil attenuates monocrotaline-induced vascular endothelial damage and pulmonary arterial hypertension. PLoS ONE. 2012 Mar 30;7(3).

25. Aizawa K, Takahari Y, Higashijima N, Serizawa K, Yogo K, Ishizuka N, et al. Nicorandil prevents sirolimus-induced production of reactive oxygen species, endothelial dysfunction, and thrombus formation. Journal of Pharmacological Sciences. 2015;127(3):284–91.

26. Liou JY, Hong HJ, Sung LC, Chao HH, Chen PY, Cheng TH, et al. Nicorandil inhibits angiotensin-II-induced proliferation of cultured rat cardiac fibroblasts. Pharmacology. 2011 Apr;87(3–4):144–51.

27. Abdel-Raheem IT, Taye A, Abouzied MM. Cardioprotective effects of nicorandil, a mitochondrial potassium channel opener against doxorubicin-induced cardiotoxicity in rats. Basic and Clinical Pharmacology and Toxicology. 2013 Sep;113(3):158–66.

28. Ashour H, Elsayed MH, Elmorsy S, Harb IA. Hypothesis: The potential therapeutic role of nicorandil in COVID-19. Clinical and Experimental Pharmacology and Physiology. 2020;47(11):1791–7.

**Appendix-I**

**Proforma**

| Patient ID | Crno |
| --- | --- |
| Date of randomization |  |
| Date of RT-PCR positive/RAT/Truenat |  |
| Date of symptom onset |  |
| Name | Age |
| Sex | Mobile no |
| DOA | Time |
| DOD | Time |
| Duration of hospital stay |  |
| The interval between symptom onset to hospital admission |  |

2.**Comorbidity**

|  | Yes/no |
| --- | --- |
| DM |  |
| HTN |  |
| H/O heart failure |  |
| Dyslipidemia |  |
| CKD |  |
| CAD |  |
| Valvular heart disease |  |
| CVA |  |
| PAD |  |
| Cancer |  |
| VTE |  |
| Chronic liver disease |  |
| COPD |  |

3.**Covid 19 symptoms**

|  | Yes/no |
| --- | --- |
| Fever |  |
| Cough |  |
| Dyspnoea |  |
| Rhinorrhoea |  |
| Sore throat |  |
| Myalgia |  |
| Diarrhoea |  |
| Nausea |  |
| Vomiting |  |

**4.Examination**

|  | At admission |
| --- | --- |
| SBP |  |
| DBP |  |
| Respiratory rate |  |
| Pulse rate |  |
| Pulmonary rales |  |

5.**Spo2**

| At admission | | At discharge |
| --- | --- | --- |
| Without oxygen support | Oxygen support (face mask) |  |
|  |  |  |
| Duration of days with spo2<95% | |  |

6.**Treatment**

|  | Yes/no |
| --- | --- |
| Hydroxychloroquine |  |
| Ivermectin |  |
| Doxycyclin |  |
| Azithromycin |  |
| Remdisivir |  |
| Tocilizumab |  |
| Vit D |  |
| Vit C |  |
| Corticosteroid |  |
| LMWH |  |
| Proton pump inhibitor |  |
| Antibiotic |  |

7.**Oxygen therapy**

|  | START | | END | | Duration(days/hour |
| --- | --- | --- | --- | --- | --- |
|  | DATE | TIME (am/pm) | DATE | TIME(am/pm) |  |
| Nasal prongs |  |  |  |  |  |
| Facemask |  |  |  |  |  |
| NRM |  |  |  |  |  |
| HFNC alone |  |  |  |  |  |
| HFNC plus face mask |  |  |  |  |  |
| HFNC plus NRM |  |  |  |  |  |
| CPAP |  |  |  |  |  |
| Invasive ventilation |  |  |  |  |  |
| The total duration of oxygen therapy |  | | | |  |

8.**Investigation**

|  | At admission | 5^th^ day |  |
| --- | --- | --- | --- |
| RBS |  |  |  |
| HB |  |  |  |
| TLC |  |  |  |
| ESR |  |  |  |
| Platelet count |  |  |  |
| Total bilirubin |  |  |  |
| Direct bilirubin |  |  |  |
| AST/ALT |  |  |  |
| ALP |  |  |  |
| LDH |  |  |  |
| S. Creatinine |  |  |  |
| S. FERRITIN |  |  |  |
| Prothrombin time |  |  |  |
| D-dimer |  |  |  |
| CRP |  |  |  |
| BNP |  |  |  |
| Procalcitonin |  |  |  |
| Troponin I |  |  |  |
| CPK |  |  |  |

9.**Primary endpoint**

|  | Yes/no |
| --- | --- |
| In-hospital mortality |  |
| Duration of mechanical ventilation |  |
| Length of hospital stay | (duration) |
| ARDS |  |
| Shock |  |
| Acute kidney injury |  |
| Impaired consciousness |  |
| Delirium |  |
| Stupor |  |
| Coma |  |

10.**Safety endpoints**

|  | Yes/no |
| --- | --- |
| Hepatotoxicity |  |
| Major bleeding |  |
| Minor bleeding |  |
| Proximal muscle weakness |  |
| Myalgia |  |

**Appendix-II**

**Patient Information Leaflet and Patient Informed Consent Form**

**Study Name:** **A randomized open-label trial to evaluate the efficacy and safety of triple therapy with nicorandil, aspirin, and atorvastatin in hospitalised patients with SARS- CoV -2 infection:**

**Principal Investigator: Dr Ambudhar Sharma**

**Site Address:**

Participant Number:_______________

**Background of this research**

As you know that covid 19 pandemic is going all over the world. Covid 19 is a disease caused by the highly contagious virus SARS-CoV-2. The disease started in DEC 2019 then spread around the world. common symptoms of covid -19 include fever, cough, dyspnoea, myalgia, anosmia, dysgeusia. Most people suffering from covid 19 will improve. The disease is associated with complications like ARDS (severe ling infection) leading to severe oxygen deficiency and can lead to death. Other complications associated with covid 19are acute cardiac injury leading to heart failure, VT/VF, AMI (heart attack), acute renal failure, stroke, venous thromboembolism. Aspirin and atorvastatin and nicorandil are cardiac medication. These drugs are in use for several years with an established safety profile.

Till date, there is no known specific medication or established treatment for covid-19. These drugs are widely used all over the world due to encouraging results. There is currently very little data available on their efficacy and safety in covid-19 patients.

This document has information to help you decide if you want to participate in the study. Before you agree to take part in the study, you need to read and understand the information given here and ask the study doctor or staff any questions you may have. They will describe the study to you and take you through this patient information sheet and informed consent form (PISICF). You can also discuss the information given in this sheet with your friends, relatives as well as doctor and take advice from them.

Purpose of the study

**The research is being done to assess the efficacy and safety of atorvastatin, aspirin, and nicorandil. the study will prove the efficacy and safety of these drugs in covid 19 and their ability to prevent complications. if you give us consent then you will be enrolled as a subject in** the **study. a brief history and examination will be done. then you will be allotted one of two groups: active intervention group (will receive aspirin, atorvastatin, nicorandil) another group will receive usual care. allotment will be done randomly with the help of a computer, not by patients' or investigators' choice. like other patients not enrolled in** the **study, baseline blood test,** ECG **or** X**ray will be done. These medications will be given for 10 days or till hospital discharge whichever is later.**

**Duration**

**The duration of** the **study would be 10 days or till your discharge.**

**Confidentiality**

Information collected from all the participants will be kept confidential. The information includes your history of illness and information collected from you during the study. All your personal and health-related information will be kept very carefully. A code will be used for your details. No information which reveals your identity will be shared with anyone else. Your details will be kept confidential as per national and international rules. Only those who are part of the study, ethics committee, and health-related agencies can see the information as permitted by the information security rules of India.

**Cost of drugs and investigations**

Drugs used in the study will be provided free of cost. The blood test will be done free of cost.

**Any risk to the subject associated with the study**

Both atorvastatin and aspirin are time-tested and safe drugs. Atorvastatin has been used to lower bad cholesterol (LDL), prevent heart attack, brain attack; and aspirin to prevent heart attack, stroke and protect stents and bypass grafts in enumerable patients without any major safety concerns. Atorvastatin can very rarely cause muscle pain, muscle injury, muscle breakdown (0.005-0.01%), and liver dysfunction (0.5-1%). However, most of these sides- effects occur with prolonged use. The probability of these side-effects with 10-day use is very low. Aspirin may also result in allergies (2.5%) and major bleeding (0.05%). However, the probability at a low dose and with ten days of use is again extremely low. The side effect of nicorandil is less include headache, GIT ulceration. During the study, we will closely monitor you for these side effects and will immediately stop the drugs when necessary. Appropriate treatment will be provided at our hospital in the extremely unlikely situation that any of these side effects occur. The investigations needed for the study are benign and do not have any potential to cause harm to the patient.

**Benefits**

Your participation in the study will help us to determine the role of these drugs in improving outcomes in covid 19. This data will guide us to make a safe and effective treatment strategy for covid 19 in the future. Your participation will help us to assess the role of aspirin and atorvastatin and nicorandil in preventing complications like Acute respiratory distress syndrome (ARDS), shock (severely low blood pressure), and other COVID-19 related major complications. This information will help in creating an effective and safe management strategy for COVID-19 patients in the future.

**Compensation of subjects for disability or death resulting from treatment**

No compensation will be provided in the very unlikely treatment-related disability or death arising out of the study. Your participation in the study is voluntary. Your decision not to participate in the study will not affect the treatment services you may be eligible for now or in near future. you can also leave the study at any point in time. Please read and review this research and patient information form carefully before you agree to participate. You may take as much time as you need to think over it.

**Permission from officials**

This study has been approved by the institutional ethics committee of the hospital/site where the study is being conducted and other organizations involved with the study.

**Responsibilities of participants participating in the study**

- You have to get the laboratory tests done at scheduled visits
- You have to follow the advice of the study doctor.
- You have to inform your study doctor and staff in case of any adverse effect or hospitalization
- You have to answer any call from your study doctor or staff to make them aware of your health condition
- Any health-related information including any medicines you are taking should be given to the study doctor or staff.
- If you change your address, you should inform the study doctor and staff.
- You cannot participate in any other study till the time you are part of this research study

**Participants in the study can leave the study or be asked to leave the study anytime**

You are participating in the study by your own decision. You can also withdraw from the study without giving any reason or and without any risk to your ongoing treatment and without affecting your legal rights. If you decide to withdraw from the study before its completion, for your safety you must meet the study doctor for a final visit.

**Compensation for participation in the study**

You will not get any money for participating in the study.,

**Patient Informed Consent Form**

**Study Name: A randomised open label trial to evaluate the efficacy and safety of triple therapy with nicorandil, aspirin, and atorvastatin in hospitalised patients with SARS- CoV -2 infection (NAAC trial)**

**Principal Investigator: Dr Ambudhar Sharma**

Participant initial : _________________

Participantnumber : ______________________

Date of birth/Age: ___________________/ _________yrs Gender: Male/Female

Participant address:________________________________________________________________

________________________________________________________________________________

Qualification: ______________________

Occupation : Student / Selfemployed / Service / Housewife / Others(please tick as appropriate)

| **I confirm the following:** | Initials of Participant |
| --- | --- |
| I confirm that I have read and understood the Patient Information Sheet dated / /2021for the above study and had the opportunity to ask questions which were fully answered to my satisfaction. |  |
| I understand that my participation in the study is voluntary and that I am free to withdraw at any time, without giving any reason, without my medical care or legal rights being affected. |  |
| I understand and agree that the Ethics Committee and the regulatory authorities will not need my permission to look at my health records both in respect of the current study and any further research that may be conducted about it, even if I withdraw from the study. I agree to this access. However, I understand that my identity will not be revealed in any information released to third parties or published. |  |
| I agree not to restrict the use of any information or results that arise from this study provided such use is only for the scientific purpose(s). |  |
| I allow the study doctor to inform my doctor about my participation in the study and to ask for my medical history and information if necessary. |  |
| I agree to take part in the above study. |  |

**Signature/ thumb impression of the participant**:

____________________________________________________________________________

Name of participant

_________________________________________ _________________

Signature/thumb impression of participant Date

**Legally Acceptable Representative:**

_____________________________________________________________________________

Name of Legally acceptable representative (LAR)

_________________________________________

Relation to the participant

__________________________________________ ________________

Signature/thumb impression of LAR Date

**Signature of the person obtaining consent:**

_________________________________________

Name of the person obtaining the consent

_________________________________________ __________________

Signature of the person obtaining the consent Date

**Impartial witness:**

____________________________________________

Name of witness

____________________________________________________

Signature of witnessDate

Address of witness_____________________________________________________________________

_____________________________________________________________________________________
